# Association Between Maternal Smoking During Pregnancy and Birth Weight: An Appropriately Adjusted Model From the Japan Environment and Children’s Study

**DOI:** 10.2188/jea.JE20150185

**Published:** 2016-07-05

**Authors:** Kohta Suzuki, Ryoji Shinohara, Miri Sato, Sanae Otawa, Zentaro Yamagata

**Affiliations:** 1 Department of Health Sciences, Interdisciplinary Graduate School of Medicine and Engineering, University of Yamanashi, Chuo, Yamanashi, Japan; 1 山梨大学大学院総合研究部 医学域 社会医学講座; 2 Center for Birth Cohort Studies, Interdisciplinary Graduate School of Medicine and Engineering, University of Yamanashi, Chuo, Yamanashi, Japan; 2 山梨大学大学院総合研究部附属出生コホート研究センター

**Keywords:** birth weight, pregnancy, smoking, 出生体重, 妊娠, 喫煙

## Abstract

**Background:**

There has been no large nationwide population-based study to examine the effects of maternal smoking status during pregnancy on birth weight that simultaneously controlled for clinical information, socioeconomic status, and maternal weight. Thus, this study aimed to determine the association between maternal smoking status during pregnancy and birth weight, while taking these confounding factors into consideration.

**Methods:**

This study examined the first-year fixed dataset from a large nationwide birth cohort study that commenced in 2011. The dataset consisted of information on 9369 singleton infants born before December 31, 2011. Children were divided into 4 groups for statistical analysis: those born to mothers who did not smoke (NS), who quit smoking before pregnancy, who quit smoking during early pregnancy, and who smoked (SM). Multiple linear regression models were conducted for each sex to examine the association between maternal smoking status during early pregnancy and fetal growth. Birth weight was estimated using the least-squares method after controlling for covariates.

**Results:**

After controlling for potential confounding factors, maternal smoking status during pregnancy was significantly associated with birth weight. There was a significant difference in birth weight between NS and SM for both male and female infants (male infants, 3096.2 g [NS] vs 2959.8 g [SM], *P* < 0.001; female infants, 3018.2 g [NS] vs 2893.7 g [SM], *P* < 0.001).

**Conclusions:**

Using data from a large nationwide birth cohort study in Japan, we have shown that maternal smoking during pregnancy may reduce birth weight by 125–136 g.

## Abstract (ja)

**背景：:**

妊娠中の喫煙が出生体重に与える影響について、臨床情報や社会経済的状況、さらには母体の体重を同時に考慮した全国規模の研究はほとんどない。そこで、この研究ではこれらの交絡因子を考慮したうえで、妊娠中の喫煙と出生体重の関連を検討することを目的とした。

**方法：:**

2011年に開始された日本を代表する大規模な出生コホート研究の、当初1年分の固定データを用いて解析を行った。このデータは2011年12月31日以前に、単胎として出生した9369人の情報を含む。これらの出生児は母親の喫煙状況により、「喫煙なし（NS）」「妊娠前に禁煙」「妊娠初期に禁煙」「喫煙あり（SM）」の4群に分類された。男女別の重回帰モデルにより、妊娠中の喫煙と胎児の発育についての関連を検討した。最小2乗法により共変量を調整した調整後出生体重を推定した。

**結果：:**

交絡因子を調整した結果、妊娠中の喫煙は男児、女児とも出生体重と有意に関連していた。NS群とSM群における出生体重には有意な差が存在した（男児：3096.2 g (NS) vs. 2959.8 g (SM) [p < 0.001]; 女児：3018.2 g (NS) vs. 2893.7 g (SM) [p < 0.001]）。

**結論：:**

日本において全国規模の出生コホート研究のデータを用いて、妊娠中の喫煙が出生体重を125—136g減少させる可能性を示した。

## INTRODUCTION

In recent years, the “Developmental Origins of Health and Diseases” hypothesis, in addition to the established “fetal programming” and “Barker’s hypothesis”, has been suggested to clarify the mechanisms of childhood growth and development.^1^ These hypotheses describe the association between a specific path of growth, such as slow fetal growth and subsequent rapid infant growth, and the development of chronic diseases in adulthood.^1^^–^^5^ Thus, appropriate fetal growth, a crucial element of these hypotheses, is considered an important factor for the future health of an individual. Some perinatal outcomes, such as low birth weight (LBW) and intrauterine growth restriction, are considered indicators of inappropriate fetal growth.

In Japan, the incidence of LBW infants has increased. For example, 5.5% of female infants born in 1975 weighed less than 2500 g, a prevalence that increased to 10.7% in 2013.^6^ Thus, because inappropriate fetal growth might be a risk factor for poor health later in life, it is important to improve fetal growth levels in Japan.

Maternal smoking during pregnancy is one of the major causes of LBW and intrauterine growth restriction.^7^^–^^10^ Moreover, low socioeconomic status (SES),^11^^–^^13^ pregnancy-induced hypertension (PIH),^14^ pre-gestational maternal body mass index (BMI),^15^ and insufficient maternal weight gain during pregnancy^16^^,^^17^ are considered risk factors for LBW. Conversely, it has been suggested that expectant mothers with diabetes mellitus (DM) and gestational diabetes mellitus (GDM) are more likely to have macrosomic babies than those without these disorders.^18^^,^^19^

With regards to SES as a risk factor for LBW, Fujiwara et al suggested that a high level of income inequality is associated with inappropriate fetal growth. This finding was based on nationally representative data; however, they did not control for the effects of maternal smoking during pregnancy, as this information was not available.^12^ Limited data in relation to confounding factors can be a major limitation in the analysis of large sets of population-based nationally representative data.

We previously examined the association between maternal smoking status during pregnancy and fetal growth in rural Japan.^20^^,^^21^ However, there were some limitations to these studies. First, as they were based in a specific area of Japan, it is difficult to apply the results to the general population. Second, because these studies were based on population-based surveys, clinical information, such as weight gain and complications during pregnancy, was not available. Thus, it was difficult to adjust for these factors, particularly for complications during pregnancy, such as PIH, DM, and GDM.

To the best of our knowledge, there have been no large nationwide population-based epidemiological studies examining the effects of maternal smoking status during pregnancy on birth weight that simultaneously controlled for clinical information, SES, pre-gestational BMI, and maternal weight gain during pregnancy, although there was a nationwide report that utilized the Japan Perinatal Registry of the Japan Society of Obstetrics and Gynecology.^22^ Because this report was based on a hospital-based survey, the participants might not be representative of Japanese pregnant women. Thus, the present study aimed to describe the association between maternal smoking status during pregnancy and birth weight while taking these confounding factors into consideration. Our analysis utilized the first-year fixed dataset from the Japan Environment and Children’s Study (JECS), a large population-based nationwide birth cohort study that commenced in 2011.^23^

## METHODS

### Study design and participants

For JECS, pregnant women were recruited between January 31, 2011, and March 31, 2014. Eligibility criteria for participants (expectant mothers) were as follows: 1) residing in the study area at the time of recruitment; 2) expected delivery date after August 1, 2011; and 3) capable of comprehending the Japanese language and completing the self-administered questionnaire. Individuals who resided outside the study area but attended cooperating health care providers within the study area were excluded from the study. Details of the JECS project have been described in a previous article.^23^ The response rate of JECS was about 79%.^24^ The present study is based on the jecs-ag-ai-20131008 dataset, which was released in October 2013. Details of this dataset were published previously.^25^ This dataset consisted of information on 9369 singleton infants born before December 31, 2011. The mean age (standard deviation [SD]) of participants in this dataset was 31.0 (5.0) years. The mean (SD) gestational duration at their pregnancy registration was 108.6 (42.7) days. The self-administered questionnaire consisted of questions regarding maternal smoking during the second trimester of their pregnancy and other covariates. Of the mothers who participated, 7734 (82.5%) completed the questionnaire.

The JECS protocol was approved by the Institutional Review Board on epidemiological studies of the Ministry of the Environment and the Ethics Committees of all participating institutions: the National Institute for Environmental Studies, which leads the JECS; the National Center for Child Health and Development; Hokkaido University; Sapporo Medical University; Asahikawa Medical College; Japanese Red Cross Hokkaido College of Nursing; Tohoku University; Fukushima Medical University; Chiba University; Yokohama City University; University of Yamanashi; Shinshu University; University of Toyama; Nagoya City University; Kyoto University; Doshisha University; Osaka University; Osaka Medical Center and Research Institute for Maternal and Child Health; Hyogo College of Medicine; Tottori University; Kochi University; University of Occupational and Environmental Health; Kyushu University; Kumamoto University; University of Miyazaki; and University of the Ryukyus. The JECS was conducted in accordance with the Helsinki Declaration and other nationally valid regulations and guidelines.

### Data collection

Information on SES and the smoking habits of mothers and their partners during the mothers’ pregnancy was collected by questionnaire during the second trimester of pregnancy. This questionnaire also included questions about lifestyle factors (stress levels, diet, alcohol consumption, physical activity, sleeping patterns, infections, and medication use) and physical environment (heat, ionizing radiation, housing conditions, and neighborhood). Maternal anthropometric data before pregnancy and data on maternal weight gain during pregnancy; complications before and during pregnancy, including PIH, DM and GDM; history of previous pregnancy; sex of infants; birth order; and perinatal outcomes, such as birth weight and gestational duration, were also collected from medical records, which were provided by subjects’ obstetricians. BMI was used to evaluate maternal weight status and was calculated according to World Health Organization standards (body weight [kg]/height [m]^2^).

### Outcomes, exposure, and covariates

The primary outcome was birth weight. Maternal smoking status during pregnancy was assessed by the questionnaire item, “Please choose an item that best describes your current smoking status”, and was grouped as follows: “smoked”, “quit smoking during early pregnancy”, “quit smoking before pregnancy”, or “never smoked (during their lifetime)”. The following were also assessed as covariates: annual household income (<2 000 000, 2 000 000–3 999 999, 4 000 000–5 999 999, 6 000 000–7 999 999, 8 000 000–9 999 999, or ≥10 000 000 JPY), maternal age at delivery (<20, 20–24, 25–29, 30–34, 35–39, or ≥40 years), maternal weight before pregnancy (BMI <18.5, 18.5–24.9, or ≥25 kg/m^2^), maternal weight gain during pregnancy (kg), prevalence of PIH, prevalence of DM/GDM, gestational duration (days), birth order (1 or more than 1), and partners’ smoking status during pregnancy (same categories as maternal smoking).

### Statistical analysis

Infants were divided into four groups for statistical analysis: born to mothers who never smoked (NS), who quit smoking before pregnancy (QSB), who quit smoking during early pregnancy (QSD), and who smoked (SM). Analysis of variance and chi-square tests were conducted to compare covariates between each group. Multiple linear regression models were constructed to examine the association between maternal smoking status during early pregnancy and fetal growth for both sexes.

Birth weight was estimated using the least-squares method, after controlling for the above covariates. The adjusted birth weights of the QSB, QSD, and SM groups were compared with that of the reference group (NS) using the Dunnett method.

In addition, because it has been suggested that maternal smoking during pregnancy, gestational duration, and birth weight could be associated with each other, we suspected it might be difficult to control for each effect simultaneously. Therefore, as a sensitivity analysis to examine the association between maternal smoking during pregnancy and birth weight, adjusted birth weights of each group were calculated without preterm birth.

All analyses were conducted using SAS version 9.3 (SAS Institute Inc., Cary, NC, USA).

## RESULTS

Overall, 3925 (50.8%) infants were male and 2927 (37.9%) were first babies. Moreover, the number of preterm births was 424 (5.5%). The mean (SD) birth weights in male and female infants were 3054.5 (421.6) g and 2964.8 (425.1) g, respectively. In addition, minimum and maximum birth weights were 200 g and 4704 g, respectively. Table 1 describes participant characteristics with respect to maternal smoking status during pregnancy. Of the 7734 expectant mothers, 4357 (56.3%), 1885 (24.4%), 1078 (13.9%), and 414 (5.4%) did not smoke, quit before pregnancy, quit during early pregnancy, and smoked currently, respectively. The mean (SD) birth weights of infants in the NS, QSB, QSD, and SM groups were 3015.2 (426.7) g, 3028.9 (407.6) g, 3010.8 (444.4) g, and 2872.8 (422.5) g, respectively. There were significant differences between groups in partners’ smoking status, annual household income, maternal weight before pregnancy, maternal weight gain during pregnancy, maternal age at delivery, and gestational duration. Conversely, the prevalence rates of PIH and DM were not significantly different between groups.

**Table 1.** Characteristics of participants by smoking status during pregnancy

Variables	Never-smokers	Ex-smokers who quit before pregnancy	Ex-smokers who quit during early pregnancy	Current smokers	*P*-value
Participants, *n* (%)	4357 (56.3%)	1885 (24.4%)	1078 (13.9%)	414 (5.4%)	
Male infants, %	50.5	50.2	52.9	50.7	0.5^a^
Primigravida, %	40.5	30.5	44.6	26.1	<0.001^a^
Partners’ smoking status					<0.001^a^
Non-smokers, %	34.5	19.1	8.1	6.3	
Ex-smokers who quit before pregnancy, %	27.5	31.5	9.3	4.6	
Ex-smokers who quit during early pregnancy, %	1.7	2.9	7.7	0.0	
Current smokers (%)	36.3	46.6	75.0	89.1	
Annual household income, %					<0.001^a^
<2 000 000 JPY	3.9	6.1	10.6	15.2	
2 000 000–3 999 999 JPY	30.9	37.9	44.6	46.1	
4 000 000–5 999 999 JPY	34.8	33.1	28.8	24.9	
6 000 000–7 999 999 JPY	17.7	14.1	9.9	8.5	
8 000 000–9 999 999 JPY	7.7	5.1	3.4	1.9	
≥10 000 000 JPY	5.0	3.6	2.6	3.4	
Mean (SD) maternal BMI before pregnancy, kg/m^2^	21.1 (3.1)	21.5 (3.6)	21.2 (3.2)	21.7 (3.9)	<0.001^b^
Pregestational maternal weight status					0.001^a^
Underweight (BMI <18.5), %	16.2	15.1	16.6	19.3	
Normal weight (BMI 18.5–24.9), %	74.1	72.7	72.8	66.2	
Overweight (BMI ≥25.0), %	9.3	12.3	10.6	14.5	
Mean (SD) maternal weight gain during pregnancy, kg	9.6 (3.7)	9.8 (3.9)	11.9 (4.3)	10.4 (4.3)	<0.001^b^
Prevalence of pregnancy-induced hypertension, %	2.9	3.0	3.7	1.9	0.3^a^
Prevalence of diabetes mellitus or gestational diabetes, %	2.8	3.7	3.0	2.9	0.3^a^
Mean (SD) maternal age at delivery, years	31.4 (4.8)	31.6 (4.7)	29.6 (5.1)	30.0 (5.6)	<0.001^b^
Maternal age group, %					<0.001^a^
<20 years	0.5	0.4	1.6	2.2	
20–24 years	7.6	6.5	14.5	16.2	
25–29 years	26.9	26.4	34.8	31.6	
30–34 years	37.6	38.4	30.9	23.9	
35–39 years	23.0	24.1	15.5	21.7	
≤40 years	4.4	4.2	2.8	4.3	
Preterm births, *n* (%)	218 (5.0)	109 (5.8)	60 (5.6)	37 (8.9)	0.008^a^
Mean (SD) gestational duration, days	274.2 (10.9)	273.9 (10.5)	273.9 (12.7)	272.1 (13.1)	0.004^b^
Low-birth-weight infants, *n* (%)	378 (8.7)	145 (7.7)	85 (7.9)	57 (13.8)	<0.001^a^
Mean (SD) birth weight of children, g	3015.2 (426.7)	3028.9 (407.6)	3010.8 (444.4)	2872.8 (422.5)	<0.001^b^

BMI, body mass index; SD, standard deviation.

^a^*P*-value was calculated by chi-square test.

^b^*P*-value was calculated by analysis of variance.

After controlling for partners’ smoking status, annual household income, birth order of children, PIH, DM/GDM, maternal weight before pregnancy, maternal weight gain during pregnancy, maternal age at delivery, and gestational duration, maternal smoking status during pregnancy was significantly associated with birth weight (*P* < 0.001 for both sexes; Table 2).

**Table 2.** Multiple linear regression model for birth weight in male and female infants

Variables	Male (*n* = 4132)			Female (*n* = 4008)		
	
	β	SE (β)	*P*-value^a^	β	SE (β)	*P*-value^a^
			(*R*^2^ = 0.42)			(*R*^2^ = 0.41)
Intercept	−2919.6	134.2	<0.001	−2932.0	133.9	<0.001
Smoking status, vs non-smokers			<0.001			<0.001
Ex-smokers who quit before pregnancy	−7.0	12.7		12.7	13.0	
Ex-smokers who quit during early pregnancy	−27.8	16.3		−39.6	17.3	
Current smokers	−136.4	24.3		−124.6	25.0	
Primigravida	−107.0	11.1	<0.001	−117.5	11.5	<0.001
Partners’ smoking status			0.4			0.3
Ex-smokers who quit before pregnancy	−21.5	14.7		22.4	15.0	
Ex-smokers who quit during early pregnancy	−39.3	33.2		3.5	34.2	
Current smokers	−13.9	13.5		−3.8	13.8	
Annual household income, %			0.6			0.4
<2 000 000 JPY	−3.5	33.9		−51.9	33.8	
2 000 000–3 999 999 JPY	−14.0	27.5		−31.5	26.8	
4 000 000–5 999 999 JPY	−30.8	27.2		−27.3	26.8	
6 000 000–7 999 999 JPY	−8.3	28.8		−14.3	28.5	
8 000 000–9 999 999 JPY	−16.8	33.1		5.3	32.8	
Pregestational maternal weight status			<0.001			<0.001
Underweight (BMI <18.5)	−93.2	14.1		−85.5	14.7	
Overweight (BMI ≥25.0)	157.3	17.7		172.8	17.8	
Maternal weight gain during pregnancy, kg	21.4	1.4	<0.001	19.0	1.4	<0.001
Pregnancy-induced hypertension	−220.8	33.1	<0.001	−82.0	30.3	0.007
Diabetes mellitus or gestational diabetes	83.1	29.8	0.005	85.8	31.7	0.007
Maternal age group at delivery, %			0.2			0.3
<20 years	3.2	64.3		96.3	63.2	
20–24 years	−0.1	20.0		−16.4	21.1	
25–29 years	−20.5	13.2		8.9	13.5	
35–39 years	−17.0	14.0		21.6	14.4	
≥40 years	39.6	26.9		17.9	27.7	
Gestational duration, days	21.4	0.5	<0.001	21.0	0.5	<0.001

BMI, body mass index; SE, standard error.

^a^*P*-value was calculated using the *t*-test and the *F*-test.

The adjusted *R*^2^ values for the male and female models were 0.42 and 0.41, respectively. The adjusted mean birth weight was calculated using the least-squares mean method. There was a significant difference in birth weight between NS and SM among both male and female infants (male infants: 3096.2 g for NS vs 2959.8 g for SM, *P* < 0.001; female infants: 3018.2 g for NS vs 2893.7 g for SM, *P* < 0.001; Table 3). Moreover, in female infants, birth weight in the NS group appeared to be higher than in the QSD group (3018.2 g for NS vs 2978.6 g for QSD, *P* = 0.06; Table 3); however, this difference was not statistically significant.

**Table 3.** Crude and adjusted mean birth weight in male and female infants

Smoking status during early pregnancy	Male					Female				
	
	Crude mean birth weight (g)	Standard Error	Adjusted mean birth weight (g)^a^	Standard Error	*P*-value^b^	Crude mean birth weight (g)	Standard Error	Adjusted mean birth weight (g)^a^	Standard Error	*P*-value^b^
Never-smokers	3062.8	9.0	3096.2	16.6		2966.8	9.2	3018.2	16.3	
Ex-smokers who quit before pregnancy	3055.3	13.5	3089.2	18.3	0.9	3002.3	13.0	3030.9	18.1	0.7
Ex-smokers who quit during early pregnancy	3067.8	17.8	3068.4	20.0	0.2	2946.8	20.3	2978.6	20.5	0.06
Current smokers	2928.4	29.3	2959.8	27.0	<0.001	2815.6	29.0	2893.7	27.5	<0.001

^a^Adjusted for partners’ smoking status, annual household income, birth order of children, pregnancy-induced hypertension, diabetes mellitus/gestational diabetes mellitus, maternal weight before pregnancy, maternal weight gain during pregnancy, maternal age group at delivery, and gestational duration; calculated by least squares mean adjustment.

^b^*P*-value was calculated using Dunnett’s test by least squares mean adjustment.

In addition, the adjusted birth weights in each group without preterm birth followed a similar trend. There was a significant difference in birth weight between NS and SM groups among both male and female infants (male infants: 3141.8 g for NS vs 3004.6 g for SM, *P* < 0.001; female infants: 3055.5 g for NS vs 2928.0 g for SM, *P* < 0.001; Table 4).

**Table 4.** Crude and adjusted mean birth weight in male and female infants without preterm birth

Smoking status during early pregnancy	Male					Female				
	
	Crude mean birth weight (g)	Standard Error	Adjusted mean birth weight (g)^a^	Standard Error	*P*-value^b^	Crude mean birth weight (g)	Standard Error	Adjusted mean birth weight (g)^a^	Standard Error	*P*-value^b^
Never-smokers	3102.6	8.3	3141.8	17.5		3007.7	8.2	3055.5	16.4	
Ex-smokers who quit before pregnancy	3110.6	12.1	3133.8	19.2	0.9	3031.6	12.3	3069.2	18.2	0.6
Ex-smokers who quit during early pregnancy	3116.9	15.5	3109.7	21.0	0.2	2999.7	16.8	3021.1	20.7	0.14
Current smokers	2994.1	26.3	3004.6	28.4	<0.001	2875.2	24.7	2928.0	28.2	<0.001

^a^Adjusted for partners’ smoking status, annual household income, birth order of children, pregnancy-induced hypertension, diabetes mellitus/gestational diabetes mellitus, maternal weight before pregnancy, maternal weight gain during pregnancy, maternal age group at delivery, and gestational duration; calculated by least squares mean adjustment.

^b^*P*-value was calculated using Dunnett’s test by least squares mean adjustment.

## DISCUSSION

This is the first Japanese study using a dataset from a nationwide birth cohort study to examine the effects of maternal smoking during pregnancy on birth weight while controlling for clinical information, SES, pre-gestational BMI, and maternal weight gain during pregnancy.

The rate of smoking in this study was 5.2%, which was similar to the value obtained in a 2010 national survey conducted by the Ministry of Health, Labour and Welfare, other Japanese nationwide reports,^26^^,^^27^ and the local cohort study conducted by our group.^21^^,^^28^ However, the prevalence of QSB was higher than that seen in our previous study in Japan; this may be due to regional differences in smoking habits. Actually, a previous report described the regional differences of smoking habits during early pregnancy using this dataset.^25^

We confirmed a significant effect of maternal smoking during pregnancy on birth weight. In fact, birth weights in the SM group for male and female infants were 136 g and 125 g lower, respectively, than in the NS group. In addition, after controlling for the effect of preterm birth, the effect of maternal smoking on birth weight was similar to the result of the main analysis. For female infants, birth weight in the QSD group was 40 g lower than the SM group. These results are consistent with the findings of previous studies, although the differences here are smaller.^21^^,^^29^ In one study, the differences in birth weight between NS and SM among both male and female infants (male infants, 3084 g [NS] vs 2960 g [SM]; female infants, 3039 g [NS] vs 2888 g [SM]) were close to those seen our previous study.^21^ Moreover, Vardavas et al reported that smoking during pregnancy was associated with a 120- to 150-g reduction in birth weight.^29^

Mean birth weight in the QSB group was lower than that in the NS group. Particularly in female infants, birth weight in the NS group appeared to be higher than in the QSD group, although this difference was not significant. These results are inconsistent with previous studies.^30^^,^^31^ However, as the sample sizes of previous studies were small, it would be difficult to detect differences in birth weight of less than 100 g. For example, Prabhu et al reported that infants born to mothers who quit smoking during early pregnancy were approximately 50 g lighter at birth than infants of non-smoking mothers, although this difference was not significant.^30^ In addition, Fasting et al reported a 20 g difference.^31^ Therefore, the effects of a 100-g difference in birth weight on subsequent growth and development should be examined. Moreover, it is essential to determine the difference in birth weight that confers a clinically significant effect.

There are some biological explanations for the association between maternal smoking during pregnancy and fetal growth. It has been suggested that smoking in expectant mothers can lead to effects in the placenta, such as calcification,^32^ a reduction in the diameter of the chorionic villi, vasoconstriction, suppression of active amino acid uptake,^33^ and increased apoptosis.^34^^,^^35^ Maternal smoking during pregnancy can also result in reduced placental size and atrophic placental villi,^36^ significantly higher umbilical artery resistance, and reduced estimated fetal weight.^37^ Fetal growth might be affected by these placental changes.

There are certain limitations to this study. First, we used a questionnaire to measure maternal smoking status instead of using objective measurements. Moreover, although the response rate was relatively high, there might be selection bias of participants. These might lead to an underestimation of maternal smoking rates, particularly due to the questionnaire-based study design.^38^ In addition, because information on partners’ smoking status was also obtained from pregnant women using the questionnaire, there might be bias. Furthermore, information of household income might be inaccurate for similar reasons. However, other data on other variables are likely to be relatively accurate and objective, as they were collected from medical records. Finally, although the previous reports suggested regional differences of smoking habits,^25^ it was difficult to examine the regional differences because the numbers of participants in each unit center of JECS were relatively small.

Despite these limitations, there has been no other nationwide community-based study examining the effect of smoking status during pregnancy on fetal growth while simultaneously controlling for confounding factors that may have influenced the results of previous studies, such as clinical information, SES, pre-gestational BMI, and maternal weight gain during pregnancy. Therefore, this study provides valuable evidence to support the importance of cessation of maternal smoking before and during pregnancy.

In conclusion, we have clarified the association of maternal smoking during pregnancy on birth weight using preliminary data from a large nationwide birth cohort study in Japan. Our results suggest that maternal smoking during pregnancy might reduce birth weight by 125–136 g. Further studies describing the effects of maternal smoking during pregnancy on fetal growth in more detail are necessary; we plan to conduct such studies using the complete JECS dataset, which will include data on approximately 100 000 infants and their parents. In addition, it is essential that the effects of maternal smoking during pregnancy on childhood growth are examined.

## ONLINE ONLY MATERIAL

Abstract in Japanese.
