# Supplementary material for: Association Between Maternal Smoking During Pregnancy and Birth Weight: An Appropriately Adjusted Model From the Japan Environment and Children’s Study
Source: J Epidemiol. 2016 Jul 5;26(7):371–7. doi: 10.2188/jea.JE20150185 (PMC4919482; doi:10.2188/jea.JE20150185)
Supplement: Abstract in Japanese. [file je-26-371-s001.pdf]

妊娠中の喫煙と出生体重の関連：「子どもの健康と環境に関する全国調査」のデータによる適切なモデルによる検討

鈴木　孝太[1]、篠原　亮次[2]、佐藤　美理[2]、小田和　早苗[2]、山縣然太朗[1,2]、子どもの健康と環境に関する全国調査グループ

[1] 山梨大学大学院総合研究部　医学域　社会医学講座

[2] 山梨大学大学院総合研究部附属出生コホート研究センター

Abstract

背景：妊娠中の喫煙が出生体重に与える影響について、臨床情報や社会経済的状況、さらには母体の体重を同時に考慮した全国規模の研究はほとんどない。そこで、この研究ではこれらの交絡因子を考慮したうえで、妊娠中の喫煙と出生体重の関連を検討することを目的とした。

方法 : 2011 年に開始された日本を代表する大規模な出生コホート研究の、当初 1 年分の固定データを用いて解析を行った。このデータは 2011 年 12 月 31 日以前に、単胎として出生した 9369 人の情報を含む。これらの出生児は母親の喫煙状況により、「喫煙なし（NS）」「妊娠前に禁煙」「妊娠初期に禁煙」「喫煙あり（SM）」の 4 群に分類された。男女別の重回帰モデルにより、妊娠中の喫煙と胎児の発育についての関連を検討した。最小 2 乗法により共変量を調整した調整後出生体重を推定した。

結果：交絡因子を調整した結果、妊娠中の喫煙は男児、女児とも出生体重と有意に関

連していた。NS 群と SM 群における出生体重には有意な差が存在した（男児 : 3096.2 g (NS) vs. 2959.8 g (SM) [p < 0.001]; 女児：3018.2 g (NS) vs. 2893.7 g (SM) [p < 0.001]）。

結論：日本において全国規模の出生コホート研究のデータを用いて、妊娠中の喫煙が出生体重を 125—136g 減少させる可能性を示した。

キーワード：出生体重、妊娠、喫煙
